# Preparation of Natural Food-Grade Core-Shell Starch/Zein Microparticles by Antisolvent Exchange and Transglutaminase Crosslinking for Reduced Digestion of Starch

**DOI:** 10.3389/fnut.2022.879757

**Published:** 2022-04-15

**Authors:** Chaofan Wang, Kaili Qin, Qingjie Sun, Xuguang Qiao

**Affiliations:** ^1^Key Laboratory of Food Processing Technology and Quality Control in Shandong Province, College of Food Science and Engineering, Shandong Agricultural University, Tai’an, China; ^2^College of Food Science and Engineering, Qingdao Agricultural University, Qingdao, China; ^3^Institute of Nutrition and Health, School of Public Health, Qingdao University, Qingdao, China

**Keywords:** core-shell microparticles, zein, antisolvent exchange, protein crosslinking, starch digestibility

## Abstract

The purpose of this study was to slow down the digestibility of starch granules by encapsulating it in zein shells. Drop of the preformed swollen corn starch (CS) granule suspension into thermal-treated zein ethanolic solution enables antisolvent precipitation of thermal-treated zein on the surface of the preformed swollen CS granules, leading to the formation of core-shell starch/zein microparticles. Confocal laser scanning microscopy images showed that the preformed swollen CS granules were coated by thermal-treated zein shells with a thickness of 0.48–0.95 μm. The volume average particle diameter of core-shell starch/zein microparticles was 14.70 μm and reached 18.59–30.98 μm after crosslinking by transglutaminase. The results of X-ray diffraction and Fourier transform infrared spectroscopy demonstrated that an interaction occurred between the preformed swollen CS granules and the thermal-treated zein. The results for thermodynamic characteristics, pasting properties, and swelling power indicated that the compact network structure of core-shell starch/zein microparticles crosslinked by transglutaminase could improve starch granule thermal stability and resistance to shearing forces. Compared to native CS, the peak gelatinization temperatures of core-shell starch/zein microparticles increased significantly (*p* < 0.05), with a maximum value of 76.64°C. The breakdown values and the swelling power at 95°C of core-shell starch/zein microparticles significantly (*p* < 0.05) decreased by 52.83–85.66% and 0.11–0.28%, respectively. The *in vitro* digestibility test showed that the contents of slowly digestible starch and resistant starch in the core-shell starch/zein microparticles increased to ∼42.66 and ∼34.75%, respectively, compared to those of native CS (9.56 and 2.48%, respectively). Our research supports the application of food-grade core-shell starch/zein microparticles to formulate low-digestibility food products.

## Introduction

Starch is the major source of carbohydrates in the human diet and represents a staple food ingredient on people’s dining tables all over the world (1). However, cooked starch can have a negative impact on human health due to its rapidly digestible nature, and this is considered one factor responsible for the increased worldwide incidence of high-risk diseases, including obesity, diabetes, and hypertension, as well as cardiovascular and cerebrovascular diseases (2). Therefore, interest has increased in designing food products that can reduce the rate and degree of starch digestion in the human small intestine, thereby preventing rapid rises in blood glucose levels and excessive blood glucose concentrations, which are currently primary dietary problems.

Starch digestion depends on both internal and external factors. The internal factors include the type, size, crystalline structure, and amylose-to-amylopectin ratio of the starch granules (3), and these have a critical impact on the digestion of native starch. For example, native starch is resistant to enzymatic digestion, whereas cooked starch is rapidly digested, and this attribute cannot be changed by modifying the internal factors. Therefore, starch digestion must be reduced by changing the external factors, which include food matrix effects (4), physical treatment, such as starch retrogradation (5), and chemical modification, such as starch crosslinking (6).

Several studies have found that the constituents and content of the food matrix encapsulating the starch granules might protect starch granules from hydrolysis by digestive enzymes, thereby reducing starch digestion (4). In fact, previous research has demonstrated that the interaction between starch and other nutrients (e.g., proteins) could significantly mitigate starch digestibility and, in turn, the glycemic response (7). Clinical studies have shown that proteins and their hydrolysates might reduce starch digestion by forming complexes with starch to lower blood glucose levels and ameliorate insulin resistance (8).

However, the current methods used to prepare starch-protein complexes mostly involve simple blending and result in complexes with low adsorption and poor stability (9, 10). In addition, naturally occurring exogenous protein tends to adsorb weakly to the surfaces of starch granules due to the thermodynamic incompatibility of starch and protein, rather than forming compact network structures that would protect the starch granules from hydrolysis by digestive enzymes. One method to overcome this limitation is to expose hydrophilic groups (e.g., asparagine) (8, 11, 12) by thermal treatment (≥90°C) to facilitate the interaction between thermal-treated protein and starch granules. This possibility triggered our interest in seeking a facile method for the fabrication of starch-protein complexes with strong interaction and stability and in determining the potential of these complexes for slowing down starch digestibility in starchy foods. Our approach was to form core-shell starch/protein microparticles.

Core-shell microparticles have wide applications in a variety of industries, including foods (13), pharmaceuticals (14), and biomedicines (15), due to their capacity for enhancing the physicochemical stabilities of the core materials and controlling their release kinetics (16). Hu et al. (17) reported the fabrication of core-shell polysaccharides/zein microparticles, which was used to encapsulate and controlled release of vitamin B6. Starch-based core-shell microparticles, such as native corn starch (CS)/zein microcapsules (18) and rice starch/bamboo shoot dietary fiber microparticles (19), have also been reported. Notably, the protein or dietary fiber shells inhibited the swelling of the starch granules, thereby restricted the access of digestive enzymes to the starch granule and reduced the rate and degree of starch digestion. However, the preparation of low digestible starch core-shell microparticles in the form of single granules encapsulated in food matrixes, such as polysaccharides or proteins, has not yet been reported.

In the present study, we aimed to develop a food-grade, biocompatible core-shell starch-protein microparticles that inhibit starch digestibility. The microparticles were fabricated using antisolvent exchange and protein crosslinking. Zein, an FDA-approved generally recognized as safe (GRAS) material, is thermally treated and used as the shell material due to its excellent film-forming and hydrophobic properties (20). The core-shell starch/zein microparticles were formed simply by dripping the preformed swollen CS granule suspension into a 70% aqueous ethanol solution containing the thermal-treated zein shell material. Solvent exchange occurs at the surface of the swollen CS granules, resulting in a gradual antisolvent exchange of the thermal-treated zein around the swollen CS granules and the formation of clearly defined core-shell microparticle structures.

In this study, transglutaminase (TGase) was used to strengthen the network structure of thermal-treated zein shells. A further study was conducted on the impact of the thermal-treated zein shells on the digestion of the starch in the core-shell microparticles. This was accomplished by performing an *in vitro* simulated digestion procedure to compare the digestibility of native CS and a series of core-shell starch/zein microparticles formed using different crosslinking periods. We further explored the mechanisms by which the thermal-treated zein shells decreases the starch digestibility in the core-shell microparticles. The findings are expected to facilitate the development of health products containing low-digestible starch.

## Materials and Methods

### Materials

Corn starch was kindly provided by Zhucheng Xingmao Corn Developing Co., Ltd. (Weifang, Shandong, China). Zein from corn (Z3625), rhodamine B, pancreatin (10 mg/mL, from porcine pancreas, 8 × USP), and α-glucosidase (from *Aspergillus niger*, 260 U/mL) were purchased from Sigma-Aldrich Chemical Co. (St. Louis, MO, United States). Calcium chloride (CaCl_2_, anhydrous, 98%) was ordered from Sinopharm Chemical Reagent Co., Ltd. (Shanghai, China). TGase (1000 U/g) was provided by Beijing Solarbio Science & Technology Co., Ltd. Nile blue was purchased from Macklin Reagent Co., Ltd. (Shanghai, China). Ethanol was obtained from Fuyu Fine Chemical Engineering Company (Tianjing, China). All other chemicals and reagents were of analytical grade. Milli-Q water was used in all experiments.

### Fabrication of Core-Shell Starch/Zein Microparticles

The preformed swollen CS granules were prepared using the method of Wang et al. (21), with minor modifications. The parameters were determined by preliminary tests, and the optimum swelling results were obtained under the following experimental conditions. Briefly, 5 g starch suspension (3 g native CS) was added to 65 mL stock solution (4.16 M CaCl_2_). The mixture was stirred at 46°C with a thermostatic magnetic stirrer (DF-101SZ, Gongyi, China) at 250–300 rpm for 1 h to swell the CS granules. The mixture was then combined with 200 g Milli-Q water, and the CS granules were left for 20 min at room temperature to absorb more water. The mixture was centrifuged at 3,600 × *g* for 5 min to obtain the preformed swollen CS granules. The preformed swollen CS granules were washed with Milli-Q water and centrifuged at 3,600 × *g* for 5 min at least three times until the CaCl_2_ was completely removed. Milli-Q water was then added to the preformed swollen CS granules until the total volume of the preformed swollen CS granule suspension was 30 mL.

Thermal pre-treatment of zein consisted of dispersing 1% w/v zein in 100 mL 70% aqueous ethanol and heating at 95°C for 30 min. To stimulate the antisolvent exchange of thermal-treated zein, 30 mL of the preformed swollen CS granule suspension was dripped into 45 mL of the thermal-treated zein ethanolic solution. The mixture was stirred at 100 rpm, and the antisolvent exchange process was normally completed in 2–3 h. The obtained core-shell starch/zein microparticles were washed and centrifuged at 3,600 × *g* for 5 min at least three times until ethanol and unadsorbed thermal-treated zein were completely removed.

**FIGURE 1:**
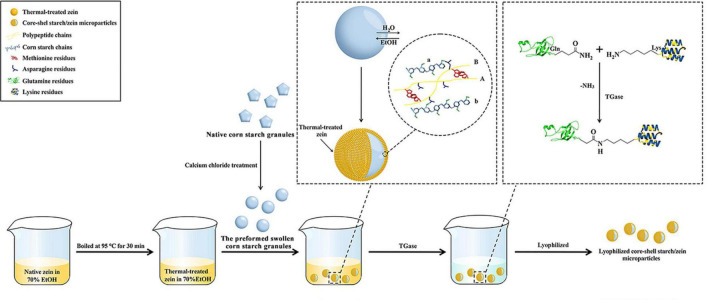
A scheme of the fabrication of core-shell starch/zein microparticles accessed by using antisolvent exchange and protein crosslinking.

Crosslinking was performed by using the method already published in literature (22, 23), with minor modifications. TGase (4.0% based on protein weight) were first dissolved in 25 mL 38°C Milli-Q water, and the dissolved enzyme was mixed with the core-shell starch/zein microparticles and incubated at 38°C. After different crosslinking periods (0, 1, and 2 h), the core-shell microparticles were resuspended in 40% ethanol and centrifuged at 3,600 × *g* for 5 min in a centrifuge (H1850, XiangYi, China). The supernatant was decanted to remove weakly adsorbed thermal-treated zein. The core-shell starch/zein microparticles were then lyophilized and designated CS-zein 0h microparticles, CS-zein 1h microparticles, and CS-zein 2 h microparticles, for the microparticles formed after 0, 1, and 2 h of crosslinking. Unshelled swollen CS granules was set as one of the control groups, where CS went through all the steps in Figure 1 but without the presence of zein.

### Particle Morphology

#### Optical Microscopy

An aqueous suspension of native CS or unshelled swollen CS granules or core-shell starch/zein microparticles was placed on a clean glass slide and covered with a coverslip, avoiding air bubble formation. The morphological structures of the microparticles were viewed with an optical microscope (Optec CCD TP510, United States) with a 40× objective.

#### Confocal Laser Scanning Microscopy

The thermal-treated zein component was stained with Nile blue (1 × 10^–3^ parts of 1% fluorophore in aqueous solution) by combining 2 g core-shell starch/zein microparticles and 25 mL aqueous Nile blue solution. After reaction for 1 min, the core-shell starch/zein microparticles were washed and centrifuged at 3600 × *g* for 5 min at least three times until the unreacted Nile blue was completely removed. All samples were wrapped in foil and stored at 4°C in a refrigerator until used. Confocal laser scanning microscopy (CLSM) images of the microparticles were acquired using an inverted laser-scanning confocal microscope (Tcorn starchsp5II, Leica, Germany) with a 10× objective and excitation at 633 nm.

The cooked lyophilized core-shell starch/zein microparticles that had been digested for 0, 20, and 120 min were also observed with the same CLSM. Before observation, the digested core-shell starch/zein microparticles were washed with copious amounts of Milli-Q water, vacuum filtered at least three times, and lyophilized to inhibit digestive enzyme activity. The shell thickness of the core-shell starch/zein microparticles was determined using the software included with the microscope. The shell thickness was obtained at different places of the shell (>3 for a single core-shell starch/zein microparticle) and over a sufficient number of core-shell starch/zein microparticles (>100).

#### Scanning Electron Microscopy

The morphologies of native CS and core-shell starch/zein microparticles were observed by field-emission scanning electron microscopy (SEM) (JSM-7500F, JEOL, Japan). The cooked core-shell starch/zein microparticles were digested for 0, 20, and 120 min and observed with the same SEM instrument. Before observation, the digested core-shell starch/zein microparticles were washed with copious amounts of Milli-Q water, vacuum filtered at least three times, and lyophilized to inhibit digestive enzyme activity. The lyophilized samples were mounted on an aluminum stub with double-sided tape and sputtered with gold for SEM imaging at 15 kV. Images were captured under 3,000× magnification.

### Particle Size Distribution

The particle size distributions of the native CS, unshelled swollen CS granules, and core-shell starch/zein microparticles were determined by dynamic light scattering (DLS) (Nano-ZS-90, Malvern Instruments Ltd., United Kingdom). The samples at a concentration of 0.1% were ultrasonically (200 W, 10 s) dispersed and evaluated at room temperature.

### Analysis of Starch and Protein Content in Core-Shell Starch/Zein Microparticles

A Megazyme Total Starch kit was used to determine the total starch content of the samples (24). A nitrogen analyzer (Kjeltec™8000MI, FOSS, Denmark) was used to determine the total protein content of the samples. A conversion factor of 6.25 was used to convert the nitrogen value to the protein content.

### Structural Properties

#### X-Ray Diffraction

The crystal structures of native CS, unshelled swollen CS granules, and core-shell starch/zein microparticles were determined by X-ray diffraction (XRD; D8 QUEST, Bruker, Germany) using Cu Kα radiation (λ = 1.5405 nm) with a divergence slit of 0.38 mm, generator voltage of 40 kV, and tube current of 40 mA. The scanning range was 4–40°, the scanning rate was 2°/min, and the step size was 0.01. The relative crystallinity of native CS and core-shell microparticles was obtained using a computerized spectrum analyzer. The relative crystallinity (RC) was calculated by the ratio of the crystalline area (Ac) to the entire area (Ae) according to the following equation:


(1) RC(%)=Ac/Ae×100%

#### Fourier Transform Infrared Spectroscopy

Structural investigations were performed using a NEXUS-870 spectrometer (Antaris II, ThermoFisher Scientific Inc., United States) with the wavenumber range of 4,000–400 cm^–1^ and a resolution of 4 cm^–1^ to record the Fourier transform infrared (FTIR) spectra of native CS, unshelled swollen CS granules, and core-shell starch/zein microparticles.

### Physical Properties

#### Thermal Properties

The thermal characteristics of the native CS, unshelled swollen CS granules, and core-shell starch/zein microparticles were determined by differential scanning calorimetry (DSC; Mettler Toledo, Schwerzenbach, Switzerland). Hermetic aluminum pans were filled with about 4.00 mg of dried microparticles and 8 μL of Milli-Q water. The samples were equilibrated overnight at room temperature and then heated from 25 to 125°C at 10°C/min. The thermal transition parameters, including onset (To), peak (Tp), and conclusion (Tc) temperatures and enthalpy change (ΔH) were determined using the data recording software. ΔH was calculated using the dry starch weight.

#### Pasting Properties

A Rapid Visco Analyzer (RVA-Tec Master, Perten, Sweden) was used to determine the pasting performance of the native CS and core-shell starch/zein microparticles by adding 3 g (dry basis, db) sample to 25 mL Milli-Q water (i.e., 10.71%, w/w, db), equilibrating the resulting slurry at 50°C for 1 min, heating from 50 to 95°C in 4 min 20 s, maintaining at 95°C for 2 min, cooling to 50°C in 4 min 20 s, and maintaining at 50°C for 1.5 min. The analyzer paddle was programmed to spin at 960 rpm for the first 10 s and 160 rpm for the rest of the test. The pasting parameters, including the peak viscosity, trough viscosity, final viscosity, breakdown, setback, and peak time were evaluated.

#### Water Solubility and Swelling Power

The water solubility (WS) and swelling power (SP) measurements were made at temperatures ranging from 50 to 90°C, with 10°C intervals using sample suspensions consisting of 200 mg (db, precision: 0.1 mg) sample and 20 mL Milli-Q water The suspensions were maintained in a water bath at each temperature for 30 min with constant shaking. The suspensions were cooled to room temperature in an ice bath, centrifuged at 3,000 × *g* for 15 min (H1850, XiangYi, China), and the supernatant was carefully decanted onto a glass plate with a specified weight (precision: 0.1 mg), dried to a constant weight at 105°C, and weighed. The residue was weighed for SP determination. The following formulas were used to determine WS and SP:


(2) WS(%)=A/S×100

(3) SP⁢(g/g)=(B×100)/S×(100-WS)

where S is the weight of the dried microparticles, A is the weight of the dried supernatant, and B is the weight of the microparticles in the swollen state.

### *In vitro* Digestibility

The *in vitro* digestion test was carried out according to Englyst et al. (25), with slight modifications. Briefly, 3 g pancreatin was dispersed in 20 mL of Milli-Q water for 5 min, 15 mL of the dispersion was decanted into a centrifuge tube, and 1.1 mL α-glucosidase was added, followed by 200 mg native CS or core-shell starch/zein microparticles and 18 mL sodium acetate buffer (pH 5.20) were added to the centrifuge tube, the suspension solution was heated in a boiling water bath for 30 min. After cooling to 37°C, 2 mL mixed enzyme solution was added and the tubes were incubated at 37°C with constant shaking. At 0, 20, 40, 60, 80, 100, and 120 min, 0.1 mL of the reaction mixture was removed, mixed with 0.9 mL 90% ethanol to deactivate the enzymes, and centrifuged at 4,000 × *g* for 10 min. The quantity of glucose in 0.l mL of the supernatant was determined using a glucose oxidase/peroxidase (GOPOD) kit (Megazyme, Ireland) and following the manufacturer’s instructions. Digestograms were created by graphing the percentage of digested starch as a function of hydrolysis time. The following equation was used to compute the content of rapidly digestible starch (RDS), slowly digestible starch (SDS), and resistant starch (RS):


(4) RDS(%)=(G20-G0)×0.9/C×100

(5) SDS(%)=(G120-G20)×0.9/C×100

(6) RS(%)=(1-RDS-SDS)×100

where G20 and G120 were the quantities of glucose (mg) released after 20 and 120 min of enzymatic hydrolysis, respectively, and C was the starch content (mg) in the sample.

### Statistical Analysis

All data were statistically evaluated and presented as mean values ± standard deviations (SD) of three replicates for each sample using SPSS 22.0 (IBM Corporation, Chicago, IL, United States). Analysis of variance (ANOVA) and Duncan’s multiple range tests were used to establish statistical significance at a level of 0.05 (*p* < 0.05).

## Results and Discussion

### Fabrication Process of Core-Shell Starch/Zein Microparticles

The schematic representation of the fabrication process for core-shell starch/zein microparticles is shown in Figure 1. The preformed swollen CS granules were prepared beforehand by treatment with CaCl_2_ (21), which interacted with the starch molecules and enhanced their SP value, resulting in partial gelatinization of the granules (26). Ca^2+^ can generate coordination compounds with –OH groups on the starch molecules, weaken the intermolecular and intramolecular hydrogen bonds within the starch structure, and promote starch granule decomposition (27, 28). The starch granules after CaCl_2_ solution treatment had a loose structure and therefore readily absorbed water and persisted in swollen form (29). The moisture content of the CaCl_2_-treated CS granules was 51.13%, which might not be sufficient to stimulate the antisolvent exchange of thermal-treated zein. Therefore, the moisture content of CaCl_2_-treated CS granules was increased by incubating them in 200 g Milli-Q water for 20 min at room temperature. This resulted in swollen CS granules with a moisture content of 58.41%. Sufficient Milli-Q water was added to the preformed swollen CS granules to bring the volume to 30 mL.

The antisolvent exchange of thermal-treated zein from 70% ethanol was induced by the addition of the preformed swollen CS granule suspensions. Solvent exchange occurred at the surface of the preformed swollen CS granules, where water diffused from the inside of the starch granules and ethanol diffused into the granules. At the surface of the preformed swollen CS granule, the outflowing water acts as an antisolvent for the thermal-treated zein, reducing the ethanol level and decreasing the solubility of the hydrophobic thermal-treated zein, causing it to assemble on the granule surface and form a shell around the hydrophilic starch granule (Figure 1). Hydrophilic groups (such as asparagine) that are ordinarily hidden within the zein inner core are exposed after cooking or boiling treatments and could facilitate the interactions between the preformed swollen CS granules and thermal-treated zein (8, 12).

The possible interactions occurring between the preformed swollen CS granules and thermal-treated zein are schematically illustrated in Figure 1. The polypeptide chains (yellow) of thermal-treated zein interact with CS chains (light blue), with asparagine residues (dark blue) on polypeptide chains A and B serving as binding sites and creating polar interactions with starch chains a and b (light blue) (8). Polypeptide chains A and B connect via methionine residues (red), allowing the thermal-treated zein to coat the surface of the swollen starch matrix. In addition, polypeptide chains (A and B) connect the two starch chains (a and b) via asparagine residues (dark blue). This might happen when thermal-treated zein becomes embedded within the interior of the preformed swollen CS granules (8).

Crosslinked solutions were made by adding 25 mL TGase solution to the core-shell starch/zein microparticles. TGase acts on the thermal-treated zein shell to increase the compactness of the network structure of thermal-treated zein, making it resistant to enzymatic degradation and water infusion (30). As shown in Figure 1, TGase catalyzes the crosslinking of thermal-treated zein via acyl transfer reactions between the γ-carboxamide group of glutamine (Gln) residues and the ε-amino group of lysine (Lys) residues, resulting in the creation of intermolecular and intramolecular isopeptide bonds (30). After different crosslinking periods (1 and 2 h), the network structure of the thermal-treated zein shells was strengthened. As a result, the preparation of the preformed swollen CS granules, as well as the subsequent antisolvent exchange and protein crosslinking, could be conducted with biocompatible and food-grade agents and materials.

### The Morphologies of Core-Shell Starch/Zein Microparticles

**FIGURE 2:**
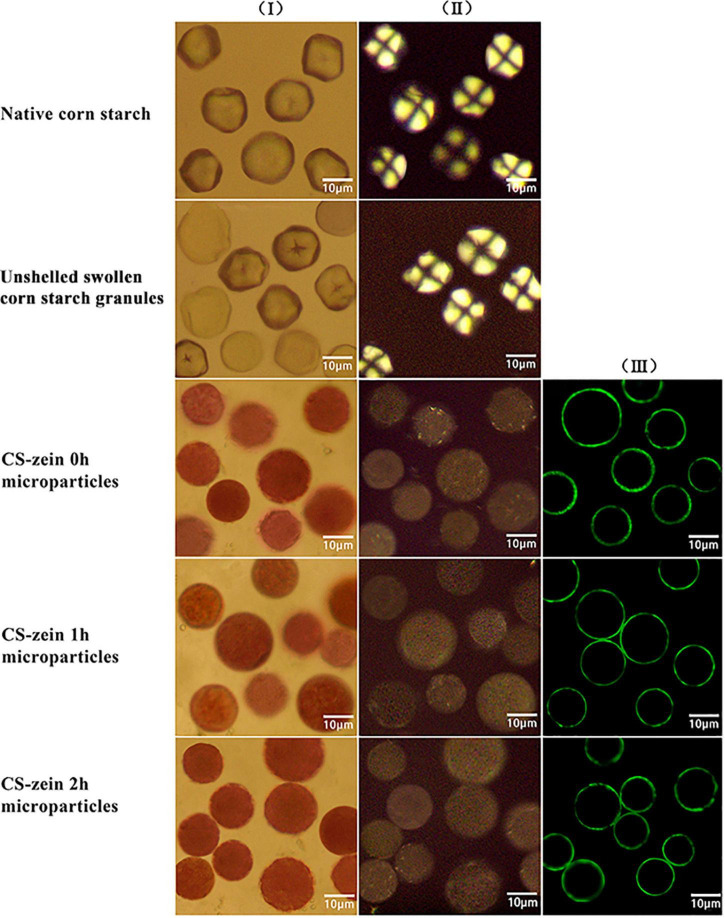
Native corn starch (line I), unshelled swollen corn starch granules (line II) and core-shell starch/zein microparticles crosslinked by TGase for 0 h (line III), 1 h (line IV), and 2 h (line V). Optical microscopy images (columns I and II); confocal scanning laser microscopic images (column III). It should be noted that not all of the images in each line were taken on the identical microparticles.

The optical microscopy images and CLSM images of the samples are shown in Figure 2. Native CS granules (line I) showed a polygonal structure, clear edges (column I), and a characteristic polarization cross (column II), the “Maltese cross,” centered at the hilum, indicating a highly ordered structure of the starch granules (31). Some unshelled swollen CS granules (line II) exhibited larger polygonal structures and blurred edges (column I) and lacked the typical polarized cross (column II), while others still maintained the optical morphology and the typical polarized cross (column II), indicating partial gelatinization of the unshelled swollen CS granules. The core-shell starch/zein microparticles (lines III, IV, and V) had an approximately spherical shape (column I) when viewed by optical microscopy and became almost opaque, with the typical polarized cross difficult to observe under polarized light (column II). This is because of the antisolvent exchange and the formation of a firm and compact thermal-treated zein shell on the surface of the preformed swollen CS granules. The thermal-treated zein shells appeared uniform and clearly defined under CLSM (column III) and were around 0.48–0.95 μm in diameter. Compared with CS-zein 0h microparticles, the CS-zein 1h microparticles and CS-zein 2h microparticles were aggregated due to the crosslinking effect of TGase, which acted on the thermal-treated zein shells of several adjacent core-shell microparticles and catalyzed the creation of intermolecular and intramolecular isopeptide bonds (30).

**FIGURE 3:**
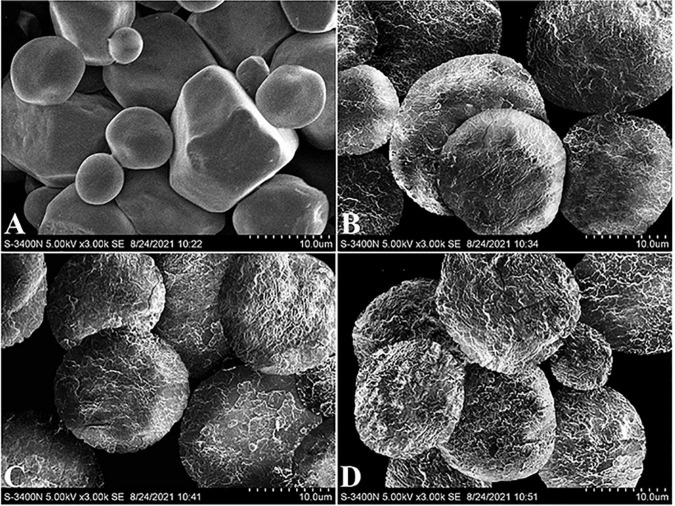
Representative SEM images of native corn starch **(A)** and core-shell starch/zein microparticles crosslinked by TGase for 0 h **(B)**, 1 h **(C)**, and 2 h **(D)**.

The morphology of native CS and core-shell starch/zein microparticles was also characterized by SEM (Figure 3). The native CS granules were polyhedral and spherical, with diameters ranging from 5 to 20 μm, whereas polygonal granules had visible edges and corners. A few depressions were found on the surface of some CS granules, in agreement with other studies (32). Compared with the native CS, the core-shell starch/zein microparticles exhibited an approximately spherical shape and a much rougher surface. The surface appeared scale-like upon careful inspection, indicating that shell formation might progress through a heterogeneous growth mechanism in which thermal-treated zein protein clusters nucleate and extend at the surface of the preformed swollen CS granules before coalescing into a continuous shell with an integrated structure (4). In contrast to our findings, Hu et al. (17) used SEM to characterize the morphology of microgel beads prepared from zein-encased hydrophilic polysaccharides and reported that the zein shell surface has a granular texture and is probably composed of aggregates of several tiny zein particles (17). The reason why their findings differed from ours might be that different core materials can lead to a different shell morphology.

### Particle Size Distribution of Core-Shell Starch/Zein Microparticles

**FIGURE 4:**
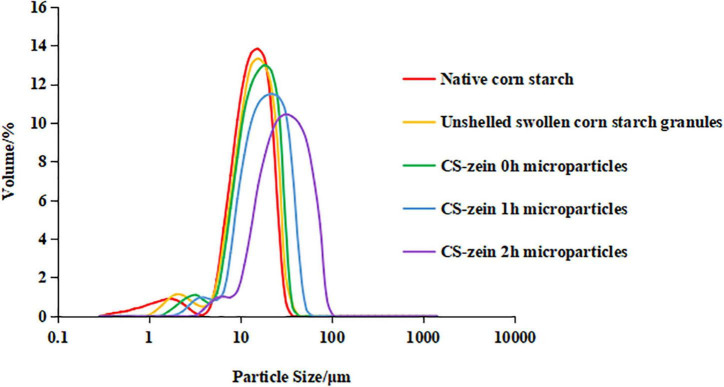
Particle size distribution of native corn starch, unshelled swollen corn starch granules, and core-shell starch/zein microparticles crosslinked by TGase for different times (0, 1, and 2 h).

As shown in Figure 4, the particle size distribution curve for the native CS, unshelled swollen CS granules, and core-shell starch/zein microparticles consisted of two different peaks, with a smaller particle size distribution of 0–10 μm and a larger particle size distribution of 10–100 μm. The volume average particle diameter d(4,3) of unshelled swollen CS granules was 13.60 μm, significantly (*p* < 0.05) higher than those of the native CS (12.44 μm), which could be caused by water permeation during the proceeding of starch gelatinization. The range of particle size of unshelled swollen CS granules was 3.89–37.00 μm, which was wider than those of the native CS (3.89–31.11 μm) for the same reason. As shown in Table 1, the volume average particle diameter d(4,3) of CS-zein 0h microparticles were 14.70 μm, which was significantly (*p* < 0.05) higher than those of the native CS (12.44 μm) and unshelled swollen CS granules (13.60 μm) and increased with the increasing crosslinking time. When the crosslinking period was 2 h, the volume average particle diameter d(4,3) of core-shell starch/zein microparticles increased to 30.98 μm. The range of particle size of of core-shell starch/zein microparticles before crosslinking began was 4.62–37.00 μm, which was wider than those of the native CS (3.89–31.11 μm) and broadened with the increasing crosslinking time. In addition, we discovered that the particle diameter of core-shell starch/zein microparticles at the peak shifted to a larger size with increasing crosslinking time. This was possibly a result of aggregation of several adjacent core-shell starch/zein microparticles in response to the crosslinking effect of TGase. This result is consistent with the evidence in the microscopy images.

**TABLE 1:** The pertinent parameter of particle size distribution of native corn starch, unshelled swollen corn starch granules, and core-shell starch/zein microparticles crosslinked by TGase for different times (0, 1, and 2 h).

Sample	d(4,3)/μ m	d(3,2)/μ m	d(0.1)/μ m	d(0.5)/μ m	d(0.9)/μ m	Range of particle size/μ m
Native corn starch	12.44 ± 0.06^e^	6.82 ± 0.04^e^	5.74 ± 0.07^e^	12.03 ± 0.08^e^	20.39 ± 0.03^e^	3.89–31.11
Unshelled swollen corn starch granules	13.60 ± 0.05^d^	9.03 ± 0.06^d^	6.16 ± 0.08^d^	12.87 ± 0.07^d^	22.38 ± 0.06^d^	3.89–37.00
CS-zein 0h microparticles	14.70 ± 0.05^c^	10.62 ± 0.06^c^	6.67 ± 0.09^c^	13.92 ± 0.03^c^	24.34 ± 0.09^c^	4.62–37.00
CS-zein 1h microparticles	18.59 ± 0.09^b^	13.37 ± 0.09^b^	8.04 ± 0.04^b^	17.03 ± 0.11^b^	31.85 ± 0.05^b^	5.50–52.33
CS-zein 2h microparticles	30.98 ± 0.03^a^	21.24 ± 0.08^a^	110.96 ± 0.06^a^	27.47 ± 0.05^a^	56.35 ± 0.04^a^	6.54–88.00

*Values indicate means ± SD of three replicates. Data in the same column with different letters are substantially different (p < 0.05).*

### Analysis of Starch and Protein Content in Core-Shell Starch/Zein Microparticles

To stimulate the antisolvent exchange of thermal-treated zein, 30 mL of the preformed swollen CS granule suspension was dripped into 45 mL of the thermal-treated zein ethanolic solution, which contained 0.42 ± 0.01 g thermal-treated zein, which is the totality of the thermal-treated zein used in this experiment. However, not all of the thermal-treated zein adsorbed on the surface of the preformed swollen CS granules. After antisolvent exchange process, the obtained core-shell starch/zein microparticles were washed and centrifuged at 3,600 × *g* for 5 min at least three times until ethanol and unadsorbed thermal-treated zein were completely removed. The supernatant after centrifugation approximately contained 0.06 g thermal-treated zein, which approximately accounted for 14.28% of the totality of the thermal-treated zein used in this experiment. The dry weight of the core-shell starch/zein microparticles before crosslinking began (i.e., CS-zein 0h microparticles) was 1.68 ± 0.11 g, the protein content of which was 21.43 ± 0.19%. Thus, the core-shell starch/zein microparticles before crosslinking began approximately contained 0.36 g thermal-treated zein, which approximately accounted for 85.71% of the totality of the thermal-treated zein used in this experiment. Namely, before crosslinking began approximately 85.71% of the totality of the thermal-treated zein used in this experiment adsorbed on the surface of the preformed swollen CS granules.

The total starch and protein contents of native CS and core-shell starch/zein microparticles are summarized in Table 2. After different crosslinking periods (0, 1, and 2 h), the protein content of core-shell starch/zein microparticles was approximately 21–27%. Increasing crosslinking periods significantly increased the protein content of the core-shell microparticles (*p* < 0.05), perhaps because TGase treatment crosslinked the weakly adsorbed zein to the strongly adsorbed zein on starch granule surface. Previous work showed that increases in added exogenous protein could reduce the rate and degree of starch digestion (33, 34).

**TABLE 2:** Total starch and protein content of native corn starch and core-shell starch/zein microparticles crosslinked by TGase for different times (0, 1, and 2 h).

Sample	Starch content/%	Protein content/%
Native corn starch	98.73 ± 0.04^a^	0.39 ± 0.11^d^
CS-zein 0h microparticles	77.80 ± 0.54^b^	21.43 ± 0.19^c^
CS-zein 1h microparticles	75.19 ± 0.78^c^	24.18 ± 0.35^b^
CS-zein 2h microparticles	71.60 ± 0.58^d^	27.84 ± 0.56^a^

*Values indicate means ± SD of three replicates. Data in the same column with different letters are substantially different (p < 0.05).*

### Structural Properties of Core-Shell Starch/Zein Microparticles

#### Crystalline Structure

**FIGURE 5:**
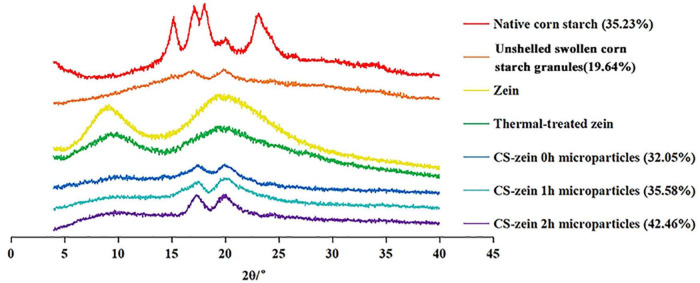
X-ray diffractogram of native corn starch, unshelled swollen corn starch granules, zein, thermal-treated zein and core-shell starch/zein microparticles crosslinked by TGase for different times (0, 1, and 2 h). The relative crystallinity is provided in parenthesis.

The XRD patterns of the native CS, unshelled swollen CS granules, zein, thermal-treated zein, and core-shell microparticles are shown in Figure 5. The native CS showed the typical A-type crystalline structure and presented main peaks at 15°, 17°, 18°, and 23° (2θ), in agreement with a previous report (35). Unshelled swollen CS granules showed a new crystalline structure with main XRD peaks at 17° and 20° (2θ). The peak at 17° 2θ indicated a B-type crystalline structure and the peak at 20° 2θ indicated the V-type polymorph. These changes could reflect changes in the molecular rearrangement of amylose and amylopectin during the decomposition and integration processes (36).

The main XRD peaks of zein were 10° and 20° (2θ), corresponding to the zein α-helix and β-sheet structures, respectively (37). However, the intensity of both peaks was decreased by thermal treatment, indicating a disruption of the intermolecular and intramolecular hydrogen bonds in the zein molecule (37).

The XRD pattern of the core-shell microparticles showed the characteristic peak at 10° (2θ) for thermal-treated zein, and its low might reflect the low protein content (38). The addition of thermal-treated zein did not change the B + V type crystalline structure of the unshelled swollen CS granules, whereas the crystallinity and the intensity of the peaks at 17° and 20° (2θ) was increased in core-shell microparticles compared to unshelled swollen CS granules. This was caused by a peak superposition due to the addition of the thermal-treated zein. Longer crosslinking periods increased the intensity and crystallinity of these peaks. The interaction between thermal-treated zein and swollen CS granules might have induced a crosslinking of the polymer chain, thereby facilitating the connection of the polymer chains and resulting in more ordered structures compared to unshelled swollen CS granules (38).

#### Fourier Transform Infrared Analysis

**FIGURE 6:**
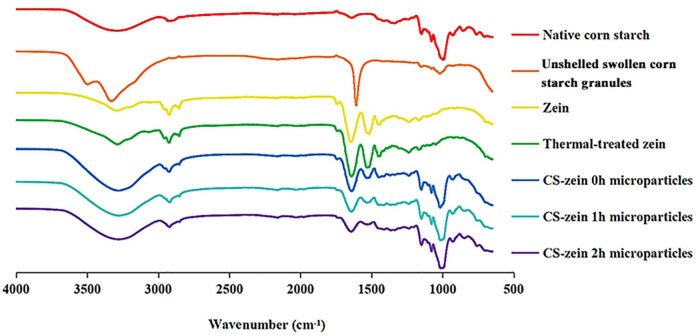
FTIR spectra of native corn starch, unshelled swollen corn starch granules, zein, thermal-treated zein and core-shell starch/zein microparticles crosslinked by TGase for different times (0, 1, and 2 h).

The FTIR spectra of native CS, unshelled swollen CS granules, zein, thermal-treated zein and core-shell starch/zein microparticles are shown in Figure 6. The native CS spectrum shows peaks at about 3,300–3,600 cm^–1^ that were related to the stretching vibration of free hydroxyl groups and intermolecular and intramolecular bound hydroxyl groups (i.e., hydrogen bonded hydroxyl groups). The stretching vibrations of intermolecular hydroxyl groups were responsible for the broad band at 3285 cm^–1^ (39). The sharp band at around 1639 cm^–1^ corresponded to bending vibrations of O–H bonds (39). Compared with the native CS, the peak wavelength of unshelled swollen CS granules was red-shifted from 3,285 to 3,330 cm^–1^, and the peak intensity increased, related to attenuation of the intensity of the intermolecular and intramolecular hydrogen bonds. Thus, unshelled swollen starch granules had a looser molecular structure that was conducive to water entry (40). The decreased intensity of the peaks at 760–1,412 cm^–1^ associated with crystallinity suggested that the ordered structure of native CS was partly disrupted (41), in agreement with the previous crystalline structure findings (Section 3.5.1).

Three characteristic zein peaks were detected at 1,640, 1,529, and 1,447 cm^–1^, associated with amide I (stretching vibration of C–O bonds), amide II (stretching vibration of C–N bonds and bending vibrations of N–H bonds), and amide III (axial bending of stretching vibration of C–N bonds), respectively (42). The intensity of the peak at 3,291 cm^–1^ was increased in the thermal-treated zein due to enhancement of the polarity by the thermal treatment (43). The intensity of peaks at 1,529 and 1,640 cm^–1^ were modestly increased, presumably indicating the presence of β-sheet structures, in agreement with previous work (37).

Fourier transform infrared analysis of the core-shell starch/zein microparticles showed an additional peaks at 1,534 cm^–1^ that were attributed to stretching vibration of C–N bonds and bending vibrations of N–H bonds (42). A new absorption peak was observed at 1,732 cm^–1^, which might be caused by the carbonyl stretching of ester (RCOOR’) bonds, indicating an interaction between the thermal-treated zein and swollen CS granules (44). Notably, the intensity of the peaks at 1,412 and 1,238 cm^–1^ was increased, which might indicate a combination of the core-shell starch/zein microparticles with water during preparation and an enhancement of the bending vibrations of C–H bonds and the stretching vibration of C=O bonds (45). Compared with the native CS, the intensity of the peak at 3,285 cm^–1^ of core-shell starch/zein microparticles increased, indicating a weakening of the intermolecular hydrogen bonds and resulting in a looser molecular structure that facilitated the entry of water into the core-shell starch/zein microparticles (40). The intensity of the peaks at 3,285, 1,639, 1,412, and 1,238 cm^–1^ decreased with increasing crosslinking time, perhaps indicating that the crosslinking effect of TGase enhanced the compactness of the thermal-treated zein layer to prevent water permeation, thereby reducing the bending vibrations of C–H bonds and the stretching vibration of C=O bonds (45).

### Physical Properties of Core-Shell Starch/Zein Microparticles

#### Thermal Properties

The thermal transition temperatures of native CS, unshelled swollen CS granules, and core-shell starch/zein microparticles are presented in Table 3.

**TABLE 3:** The onset (To), peak (Tp) and conclusion (Tc) temperatures and enthalpy change (ΔH) of native corn starch, unshelled swollen corn starch granules, and core-shell starch/zein microparticles crosslinked by TGase for different times (0, 1, and 2 h).

Sample	To/°C	Tp/°C	Tc/°C	Δ H/(J/g)
Native corn starch	65.24 ± 0.27^d^	70.02 ± 0.28^d^	74.88 ± 0.12^d^	–12.06 ± 0.07^a^
Unshelled swollen corn starch granules	50.19 ± 0.13^e^	56.76 ± 0.18^e^	63.71 ± 0.21^e^	–2.32 ± 0.17^e^
CS-zein 0h microparticles	67.61 ± 0.17^c^	71.52 ± 0.10^c^	75.73 ± 0.13^c^	–9.39 ± 0.12^b^
CS-zein 1h microparticles	69.91 ± 0.09^b^	73.42 ± 0.17^b^	77.35 ± 0.26^b^	–7.00 ± 0.09^c^
CS-zein 2h microparticles	73.57 ± 0.10^a^	76.64 ± 0.08^a^	80.24 ± 0.15^a^	–3.94 ± 0.17^d^

*Values indicate means ± SD of three replicates. Data in the same column with different letters are substantially different (p < 0.05).*

Compared with the native CS, unshelled swollen CS granules underwent easier gelatinization, as determined by the significant (*p* < 0.05) decrease in the thermal transition temperatures (To, Tp, and Tc temperatures) and ΔH of unshelled swollen CS granules. The loose structure of unshelled swollen CS granules was responsible for the decrease in energy required to unravel the helical structure; thus, the ΔH was lower than that of native CS.

The Tp of the core-shell starch/zein microparticles was significantly increased (*p* < 0.05) compared with that of the native CS, indicating that the formation of core-shell starch/zein microparticles improved the thermal stability of the native CS. Potato starch/soy protein complexes (46) and indica rice starch/whey protein isolate complexes (9) also have also shown increased starch gelatinization temperatures. This phenomenon could be caused by prevention of water permeation by the protein–starch matrix barrier and by starch–protein interactions into the crystalline starch architecture during heating; thus, a higher temperature was required to destroy the crystalline starch (33, 34). The ΔH of the core-shell starch/zein microparticles was significantly (*p* < 0.05) decreased compared with that of the native CS, and decreased with increasing crosslinking time. This probably reflected the increased compactness of thermal-treated zein shells with the increasing crosslinking time. Gelatinization of some core-shell starch/zein microparticles was difficult, and these core-shell starch/zein microparticles may not be involved in endothermic processes.

#### Pasting Properties

**FIGURE 7:**
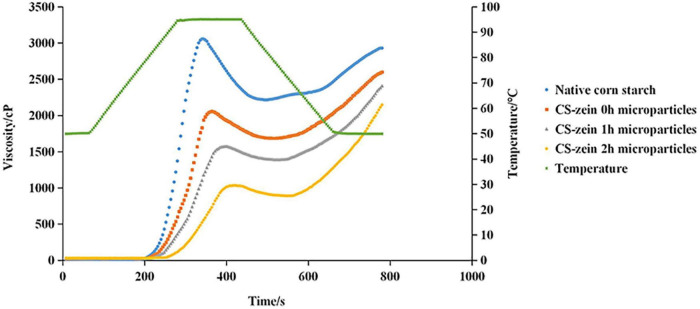
RVA patterns of native corn starch and core-shell starch/zein microparticles crosslinked by TGase for different times (0, 1, and 2 h).

The pasting profiles of native CS and core-shell starch/zein microparticles are shown in Figure 7. The viscosity increase in the core-shell starch/zein microparticles was much slower than that of the native CS, and it was further slowed with increasing crosslinking time. Table 4 displays the pasting parameters of native CS and core-shell starch/zein microparticles. The peak time was significantly (*p* < 0.05) higher for the core-shell starch/zein microparticles than for the native CS, and was further increased with increasing crosslinking time. The original buried hydrophilic groups (such as asparagine) inside the zein were gradually exposed after heating and came into contact with water molecules (43). The competition for hydration between thermal-treated zein and swollen CS granules was responsible for the higher pasting temperatures of core-shell starch/zein microparticles, as water permeation into starch granules was prevented, thereby limiting the starch granule expansion via a stereo-hindrance effect (47). Compared to native CS, the peak viscosity of core-shell starch/zein microparticles was significantly (*p* < 0.05) decreased, mainly because of the low starch content. In addition, thermal-treated zein might compete for water molecules with swollen CS granules, thereby inhibiting starch granule expansion and amylose leaching (48). The peak viscosity of core-shell starch/zein microparticles decreased with increasing crosslinking time since the protein content of the core-shell starch/zein microparticles increased during the crosslinking process.

**TABLE 4:** Pasting parameters of native corn starch and core-shell starch/zein microparticles crosslinked by TGase for different times (0, 1, and 2 h).

Sample	Peak viscosity /cP	Trough viscosity /cP	Breakdown /cP	Final viscosity /cP	Setback /cP	Peak time /min
Native corn starch	3058.33 ± 12.34^a^	2216.67 ± 9.61^a^	841.67 ± 2.89^a^	2930.00 ± 16.52^a^	713.33 ± 7.02^d^	5.67 ± 0.07^d^
CS-zein 0 h microparticles	2079.33 ± 18.90^b^	1682.33 ± 14.57^b^	397.00 ± 4.36^b^	2594.00 ± 19.97^b^	911.67 ± 5.51^c^	6.09 ± 0.10^c^
CS-zein 1 h microparticles	1573.67 ± 7.37^c^	1377.00 ± 10.82^c^	196.67 ± 5.51^c^	2392.00 ± 8.54^c^	1015.00 ± 2.65^b^	6.64 ± 0.08^b^
CS-zein 2 h microparticles	1019.67 ± 13.58^d^	899.00 ± 11.36^d^	120.67 ± 4.16^d^	2133.33 ± 11.68^d^	1234.33 ± 2.52^a^	6.98 ± 0.04^a^

*Values indicate means ± SD of three replicates. Data in the same column with different letters are substantially different (p < 0.05).*

Compared to native CS, the breakdown of core-shell starch/zein microparticles was markedly decreased by 2.12- to 11.91-fold. Increasing crosslinking time decreased the breakdown values for the core-shell starch/zein microparticles, suggesting that the thermal-treated zein shells on core-shell starch/zein microparticles might effectively protect swollen CS granules from damage by limiting starch granules expansion. As a result, thermal-treated zein shells increased the resistance of the starch granules to shearing forces. The recombination and rearrangement of leached amylose molecules is referred to as setback, and it represents the tendency of starch gels to undergo short-term retrogradation (49). The setback values were considerably (*p* < 0.05) greater for the core-shell starch/zein microparticles than for the native CS and increased with increasing crosslinking time. Thermal-treated zein might aid in the formation of the gel network structure of swollen CS granules, thereby speeding up the short-term retrogradation of swollen CS granules (50).

#### Water Solubility and Swelling Power

**FIGURE 8:**
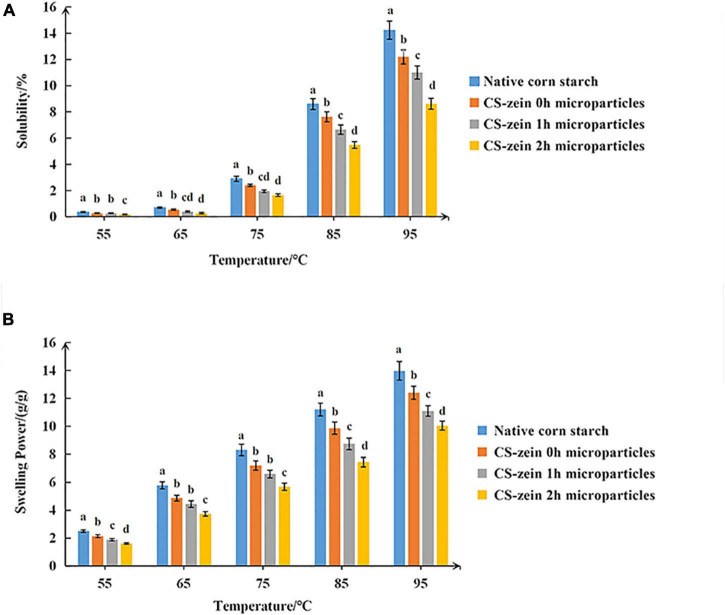
**(A)** The water solubility (WS) of native corn starch and core-shell starch/zein microparticles crosslinked by TGase for different times (0, 1, and 2 h). **(B)** The Swelling power (SP) of native corn starch and core-shell starch/zein microparticles crosslinked by TGase for different times (0, 1, and 2 h).

The WS and SP of native CS and core-shell starch/zein microparticles are shown in Figure 8. For all samples, the WS and SP increased with increasing temperature. The WS and SP in water were significantly lower for the core-shell starch/zein microparticles than for native CS. The WS and SP of core-shell starch/zein microparticles decreased with increasing crosslinking time. The decrease in WS could be explained by an effect of the compact network structure of thermal-treated zein shells formed after TGase treatment, as this would limit the permeation of water molecules. The hydrophobicity of the thermal-treated zein could also hinder the interaction between water molecules and the swollen CS granules (33, 34). Removal of endogenous proteins from starch can result in rapid swelling and promote starch digestion (51).

Thermal-treated zein might lower starch digestion through two physicochemical mechanisms: (1) the thermal-treated zein shell limits the swelling of starch granules and (2) compact thermal-treated zein shell serves as barrier between digestive enzymes and starch granules. Further investigation is required to determine the accurate physicochemical mechanisms involved.

### *In vitro* Digestibility

**FIGURE 9:**
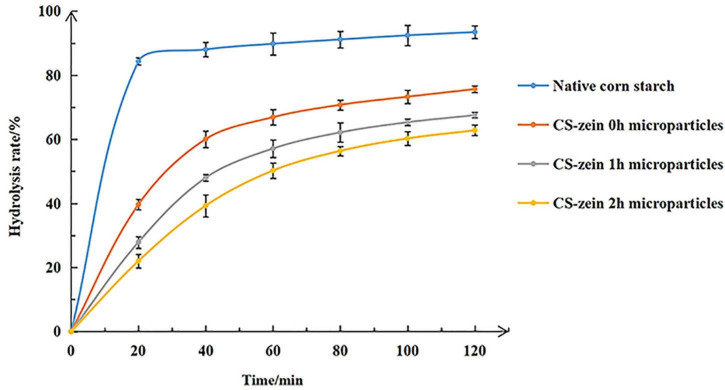
*In vitro* hydrolysis profiles of native corn starch and core-shell starch/zein microparticles crosslinked by TGase for different times (0, 1, and 2 h).

The *in vitro* hydrolysis profiles of the various cooked samples (native CS, CS-zein 0h, CS-zein 1h, and CS-zein 2h microparticles) are shown in Figure 9. After cooking, 84.29% of native CS was rapidly digested during the first 20 min, in agreement with previous studies (52). For core-shell starch/zein microparticles, the CS-zein 0h microparticles were digested slowly during the first 20 min, with a hydrolysis rate of only about 40%. The hydrolysis rate for the core-shell starch/zein microparticles decreased with increasing crosslinking time. For example, CS-zein 2h microparticles had a very slow hydrolysis rate, and only 22.01% of the starch was digested during the first 20 min. After 120 min, the hydrolysis rate of CS-zein 0h, CS-zein 1h, and CS-zein 2h microparticles was 75.64, 67.53, and 62.81%, respectively, while that of native CS was 93.44%. This decrease could indicate that the compact thermal-treated zein network and the interactions between amino acid residues and starch chains prevented the penetration of the digestive enzymes and reduced the degree and rate of starch hydrolysis (30, 34).

**FIGURE 10:**
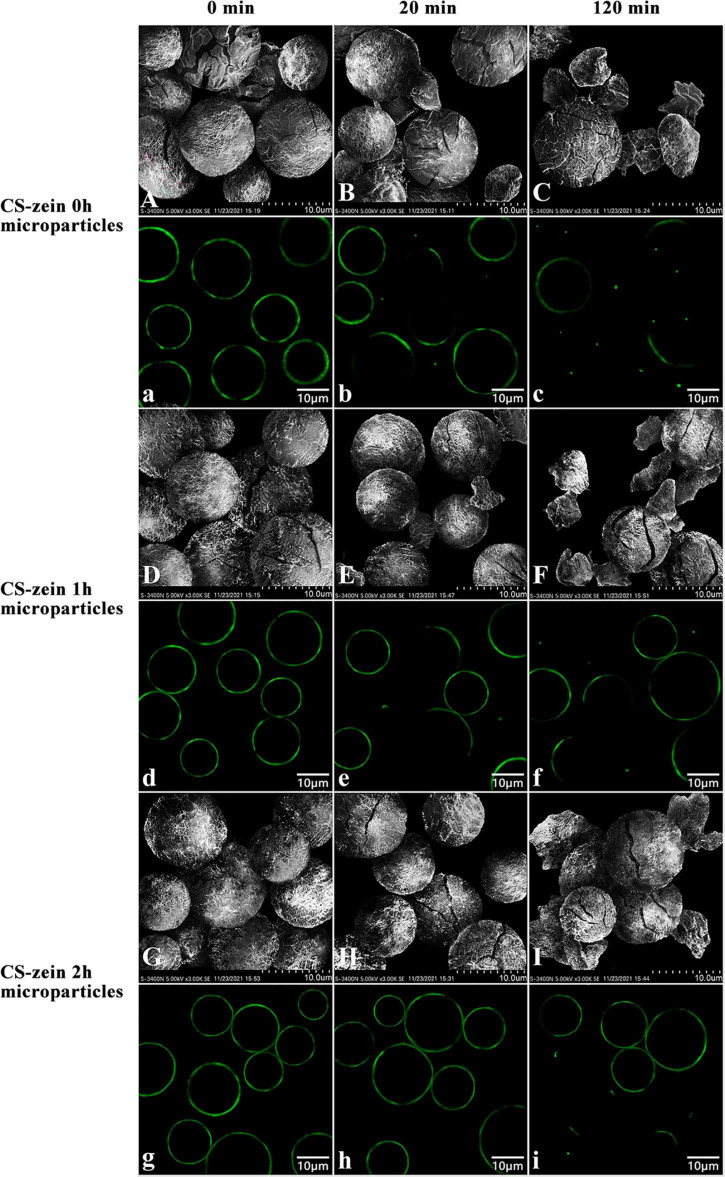
Scanning electron micrographs and confocal scanning laser microscopic images of CS-zein 0h microparticles after 0 min **(A, a)**, 20 min **(B, b)**, 120 min **(C, c)** of enzymatic hydrolysis, and CS-zein 1h microparticles after 0 min **(D, d)**, 20 min **(E, e)**, 120 min **(F, f)** of enzymatic hydrolysis, and CS-zein 2h microparticles after 0 min **(G, g)**, 20 min **(H, h)**, 120 min **(I, i)** of enzymatic hydrolysis.

The changes in core-shell starch/zein microparticles and thermal-treated zein shells at different digestion times were investigated by SEM and CLSM. Figures 10A,a,D,d,G,g, show the SEM and CLSM images of core-shell starch/zein microparticles after cooking for 30 min without enzymatic hydrolysis. Figures 10A,a show some cracks on the surface of the CS-zein 0h microparticles; these were the cracks due to breaking of the thermal-treated zein shell during cooking. The CS-zein 1h microparticles (Figures 10D,d) had fewer cracks, and the CS-zein 2h microparticles (Figures 10G,g) had no cracks, indicating that the crosslinking effect of TGase improved the thermal stability of the core-shell starch/zein microparticles, in agreement with the DSC data. The images of core-shell starch/zein microparticles digested for 20 min are shown in Figures 10B,b,E,e,H,h. The cracks on the surfaces of core-shell starch/zein microparticles increased (Figures 10B,b,E,e,H,h), and some fragments and chunks appeared (Figures 10B,b,E,e). The CS-zein 1h microparticles (Figures 10E,e) had fewer cracks, chunks, and fragments, and the CS-zein 2h microparticles (Figures 10H,h) had no chunks or fragments. Hydrolysis of the core-shell starch/zein microparticles by digestive enzymes for 120 min increased the extent of the cracks, fragments, and chunks (Figures 10C,c,F,f,I,i), indicating that core-shell starch/zein microparticles were hydrolyzed after 120 min. Longer crosslinking periods decreased the hydrolysis rate of core-shell starch/zein microparticles, in agreement with the *in vitro* hydrolysis profiles.

The RDS, SDS, and RS contents of native CS and core-shell starch/zein microparticles are summarized in Table 5. After cooking, the RDS content of native CS was as high as 87.96%, and the SDS and RS contents were 9.56 and 2.48%, respectively. For the core-shell starch/zein microparticles, the SDS and RS contents of CS-zein 0h microparticles were 37.63 and 21.05%, respectively. As the crosslinking periods increased, the RDS content decreased and the SDS and RS contents increased. A crosslinking period of 2 h increased the SDS content of core-shell starch/zein microparticles to 42.66% (a 5-fold increase) and the RS content to 34.75% (a 14-fold increase).

**TABLE 5:** *In vitro* digestibility of native corn starch and core-shell starch/zein microparticles crosslinked by TGase for different times (0, 1, and 2 h).

Sample	RDS/%	SDS/%	RS/%
Native corn starch	87.96 ± 1.12^a^	9.56 ± 2.06^c^	2.48 ± 1.96^d^
CS-zein 0h microparticles	41.32 ± 1.71^b^	37.63 ± 2.73^b^	21.05 ± 1.06^c^
CS-zein 1h microparticles	29.04 ± 1.86^c^	41.43 ± 1.01^ab^	29.53 ± 0.86^b^
CS-zein 2h microparticles	22.59 ± 2.43^d^	42.66 ± 1.12^a^	34.75 ± 1.85^a^

*Values indicate means ± SD of three replicates. Data in the same column with different letters are substantially different (p < 0.05).*

The film formed by the thermal-treated zein around the swollen CS granules prevented starch hydrolysis by the digestive enzymes. As the crosslinking periods increased, the degree of compactness of the thermal-treated zein film was improved, thereby improving the protective effect of the thermal-treated zein film. Previous studies have demonstrated that the interaction of starch with proteins in foods can significantly decrease its digestibility (7). Our research showed that exogenous proteins might have similar effects. The increased SDS and RS contents of core-shell starch/zein microparticles might be related to decreased enzyme access due to the compact thermal-treated zein shell. Chen et al. (53) also used zein to encapsulate a maize starch/maize oil complex and found that the SDS and RS content increased from 4.81% to 16.23%.

## Conclusion

This research presented a facile method to fabricate natural food-grade core-shell starch/zein microparticles, based on antisolvent exchange and protein crosslinking. The volume average particle diameter of core-shell starch/zein microparticles was 14.70 μm, and increased to 18.59–30.98 μm with a shell thickness of 0.48–0.95 μm after crosslinking by TGase. The CLSM and SEM images showed that swollen CS granules were encapsulated by thermal-treated zein shells. The results of DSC, RVA, and SP indicated that the compact network structure of core-shell microparticles crosslinked by TGase improved the thermal stability of the starch granules and their resistance to shearing forces. The CS-zein 2h microparticles showed the highest Tp, which was 6.62°C higher than the Tp for native CS. The breakdown values and the SP at 95°C for the core-shell starch/zein microparticles significantly decreased (*p* < 0.05) by 52.83–85.66% and 0.11–0.28%, respectively. During the digestion process, the thermal-treated zein shells acted as physical barriers that protected starch granules from hydrolysis by digestive enzymes. Compared to the cooked CS, the SDS and RS contents of cooked CS-zein 2h microparticles increased by about 5- and 14-fold, respectively. The research provides a new strategy for the slow digestion of starch by encapsulating starch granules in thermal-treated zein shells, which will contribute to develop low digestible health products.

## Data Availability Statement

The original contributions presented in the study are included in the article/Supplementary Material, further inquiries can be directed to the corresponding authors.

## Author Contributions

CW: writing original draft–lead, writing review and editing—supporting, and conceptualization—supporting. KQ: conceptualization—supporting; writing original draft–supporting, and investigation—supporting. QS: resources–lead, conceptualization—lead, funding acquisition—lead, supervision—lead, and writing review and editing—lead. XQ: resources—lead, conceptualization—lead, funding acquisition—lead, supervision—lead, writing review and editing—lead. All authors contributed to this work and approved the final version of the manuscript.

## Conflict of Interest

The authors declare that the research was conducted in the absence of any commercial or financial relationships that could be construed as a potential conflict of interest.

## Publisher’s Note

All claims expressed in this article are solely those of the authors and do not necessarily represent those of their affiliated organizations, or those of the publisher, the editors and the reviewers. Any product that may be evaluated in this article, or claim that may be made by its manufacturer, is not guaranteed or endorsed by the publisher.

## Supplementary Material

Supplementary Table 1 Data sheet of the particle size distribution of native corn starch, unshelled swollen corn starch granules, and core-shell starch/zein microparticles crosslinked by TGase for different times (0, 1, and 2 h).

Supplementary Table 2 Data sheet of total starch and protein content of native corn starch and core-shell starch/zein microparticles crosslinked by TGase for different times (0, 1, and 2 h).

Supplementary Table 3 Data sheet of X-ray diffractogram of native corn starch, unshelled swollen corn starch granules, zein, thermal-treated zein and core-shell starch/zein microparticles crosslinked by TGase for different times (0, 1, and 2 h).

Supplementary Table 4 Data sheet of FTIR spectra of native corn starch, unshelled swollen corn starch granules, zein, thermal-treated zein and core-shell starch/zein microparticles crosslinked by TGase for different times (0, 1, and 2 h).

Supplementary Table 5 Data sheet of the thermal parameters of native corn starch, unshelled swollen corn starch granules, and core-shell starch/zein microparticles crosslinked by TGase for different times (0, 1, and 2 h).

Supplementary Table 6 Data sheet of the pasting parameters of native corn starch and core-shell starch/zein microparticles crosslinked by TGase for different times (0, 1, and 2 h).

Supplementary Table 7 Data sheet of the water solubility (WS) of native corn starch and core-shell starch/zein microparticles crosslinked by TGase for different times (0, 1, and 2 h).

Supplementary Table 8 Data sheet of the swelling power (SP) of native corn starch and core-shell starch/zein microparticles crosslinked by TGase for different times (0, 1, and 2 h).

Supplementary Table 9 Data sheet of the pertinent parameter of *in vitro* digestibility of native corn starch and core-shell starch/zein microparticles crosslinked by TGase for different times (0, 1, and 2 h).
